# To What Extent Can Motor Imagery Replace Motor Execution While Learning a Fine Motor Skill?

**DOI:** 10.5709/acp-0197-1

**Published:** 2016-12-31

**Authors:** Jagna Sobierajewicz, Sylwia Szarkiewicz, Anna Przekoracka-Krawczyk, Wojciech Jaśkowski, Rob van der Lubbe

**Affiliations:** 1 Department of Cognitive Psychology, University of Finance and Management, Warsaw, Poland; 2 Vision and Neuroscience Laboratory, NanoBioMedical Centre, Adam Mickiewicz University, Poznan, Poland; 3 Laboratory of Vision Science and Optometry, Faculty of Physics, Adam Mickiewicz University, Poznan, Poland; 4 Institute of Computing Science, Poznan University of Technology, Poznan, Poland; 5 Cognitive Psychology and Ergonomics, University of Twente, Enschede, The Netherlands

**Keywords:** motor imagery, fine motor skill, learning, motor execution, motor preparation, DSP task, EEG

## Abstract

Motor imagery is generally thought to share common mechanisms with motor execution. In the present study, we examined to what extent learning a fine motor skill by motor imagery may substitute physical practice. Learning effects were assessed by manipulating the proportion of motor execution and motor imagery trials. Additionally, learning effects were compared between participants with an explicit motor imagery instruction and a control group. A Go/NoGo discrete sequence production (DSP) task was employed, wherein a five-stimulus sequence presented on each trial indicated the required sequence of finger movements after a Go signal. In the case of a NoGo signal, participants either had to imagine carrying out the response sequence (the motor imagery group), or the response sequence had to be withheld (the control group). Two practice days were followed by a final test day on which all sequences had to be executed. Learning effects were assessed by computing response times (RTs) and the percentages of correct responses (PCs). The electroencephalogram (EEG ) was additionally measured on this test day to examine whether motor preparation and the involvement of visual short term memory (VST M) depended on the amount of physical/mental practice. Accuracy data indicated strong learning effects. However, a substantial amount of physical practice was required to reach an optimal speed. EEG results suggest the involvement of VST M for sequences that had less or no physical practice in both groups. The absence of differences between the motor imagery and the control group underlines the possibility that motor preparation may actually resemble motor imagery.

## Introduction

 A wide range of evidence supports the idea that the learning of a motor skill (like throwing a ball, grasping an object, but also making a specific sequence of finger movements) may already occur when a person mentally simulates executing this skill ( Allami, Paulignan, Brovelli, & Boussaoud, 2008; Doussoulin & Rehbein, 2011; Sobierajewicz, Przekoracka-Krawczyk, Jaśkowski, Verwey, & van der Lubbe, 2016). This mental simulation of carrying out a motor act without making any overt body movements is often denoted as motor imagery (Jeannerod, 1995). The benefit of motor imagery for learning a motor skill may arise from the overlap between *motor imagery* and motor execution. For example, the duration of imagined actions is similar to real execution, and comparable brain areas are activated during motor execution and motor imagery (Geradin et al., 2000; Kraeutner, Gionfriddo, Bardouille, & Boe, 2014; Lotze & Halsband, 2006 ; Papaxanthis, Pozzo, Skoura, & Schieppati, 2002; Solodkin, Hlustik, Chen, & Small, 2004; Xu et al., 2014; Zhang et al., 2011). Given the resemblance between motor imagery and motor execution, and the observation that motor imagery induces motor learning, it may be argued that motor execution that is normally needed to acquire a specific skill can be partly substituted by motor imagery. In the present study, we focused on a sequence of finger movements, a so-called *fine motor skill* (Payne & Isaacs, 1987), which implies the possibility to assess sequence-specific learning effects. The main issue to be addressed concerns the extent to which learning a fine motor skill by motor imagery can replace physical practice, which was examined by employing different proportions of executed and imagined movement sequences. A control group was also included in which participants were asked to withhold executing the movement sequence rather than to mentally simulate carrying out this sequence, to establish whether the explicit instruction to perform motor imagery is needed to show sequence-specific learning effects. 

 Learning a motor sequence is reflected in increased accuracy and a reduction in the overall time needed to execute the sequence of movements. Execution of a learned sequence of movements is thought to require less effort and to reduce the need of attentional monitoring (Abrahamse, Ruitenberg, De Kleine, & Verwey, 2013; Diedrichsen & Kornysheva, 2015; Willingham, 1998). Thus, attention may be allocated to other goals, which may explain the ability of professional musicians to have a conversation while playing the piano. It has been shown that motor performance can benefit from motor imagery training. For example, previous studies reported that motor imagery leads to increased muscular force (Lebon, Collet, & Guillot, 2010; Ranganathan, Siemionow, Liu, Sahgal, & Yue, 2004; Yue & Cole, 1992), improved motor timing (Pascual-Leone et al., 1995), and motor recovery (Abbas et al., 2011; Cho, Kim, & Lee, 2013; Lee, Song, Lee, Cho, & Lee, 2011; Maillet et al., 2013; Mulder, 2007). Other studies revealed beneficial effects of motor imagery when it was combined with physical practice in the acquisition of a motor skill (Driskell, Copper, & Moran, 1994; Mulder, 2007; Page, Levine, Sisto, & Johnston, 2011; Warner & McNeill, 2013). Nevertheless, several studies revealed that improved motor performance was larger after physical training than after motor imagery training, which has been ascribed to the absence of sensory feedback during motor imagery (Gentili, Han, Schweighofer, & Papaxanthis, 2010; Gentili, Papaxanthis, & Pozzo, 2006). Likewise, the effectiveness of the combination of mental with physical practice was not as strong as physical practice alone (Debarnot, Abichou, Kalenzaga, Sperduti, & Piolino, 2015; Feltz & Landers, 1983; Hird, Landers, Thomas, & Horan, 1991; Schuster et al., 2011). 

 Allami et al. (2008) investigated to what extent different rates of motor imagery and physical practice influence visuo-motor learning by employing a grasping task. Participants were divided into five groups. The first four groups were tested with different proportions of executed versus imagined trials of a total of 240 trials, namely, 100%, 75%, 50%, and 25% of all trials. The fifth group (the control group) imagined visual rotation (180 trials) and executed the same motor task as all participants for 60 trials. The major dependent variable was movement time, which was defined as the time interval from grasping the object to inserting it into the support. Different rates of imagined/executed trials affected the total time needed to execute the task. Results revealed that groups with up to a total of 75% of imagined trials displayed similar performance as the group with full physical practice. Allami et al. (2008) concluded that a large proportion of mental practice trials combined with physical practice may lead to similar performance as physical practice alone. However, they employed a between-subjects design, which implies that initial group differences cannot be ruled out. Furthermore, learning differences due to different proportions of mental versus physical practice will be more difficult to demonstrate. In other words, the sensitivity to detect differences as a function of mental/physical practice may not have been optimal. The study of Allami et al. (2008) does not clarify what processes actually benefit from motor imagery. However, in a recent study, Allami et al. (2014) measured the electroencephalogram (EEG) with a comparable design as Allami et al. (2008). Now, one group executed all trials, whereas a second group imagined 75% of the trials. The fronto-central N2 component of the event-related potential (ERP) was examined, which occurred just before the start of the arm movement. The amplitude of the N2 component increased due to learning, and this effect was comparable for both groups. These results were taken as support for the idea that both groups learned the required task in a comparable manner. Although the N2 component has been associated with motor preparation and sensorimotor integration, some research shows that it may not be that specific for motor processes, as it has also been related with attentional processes and conflict monitoring (see Carter et al., 1998; Folstein & Petten, 2008; Kobana & Pourtois, 2014; Patel & Azzam, 2005; Ridderinkhof, Ullsperger, Crone, & Nieuwenhuis, 2004). 

 Recently, Sobierajewicz et al. (2016) used another task to study the effects of motor imagery. A Go/NoGo paradigm was employed, which was developed by De Kleine and Van der Lubbe (2011). In their task version, a sequence of finger movements with the left or right hand has to be carried out, which is signaled by a sequence of visuospatial cues. In the case of a subsequent Go signal, the sequence has to be executed while execution should be withheld after a NoGo signal. Use of this task has the advantage that sequence-specific learning effects can be assessed while controlling for unspecific learning effects, which may occur due to increased familiarity with the task procedure. Furthermore, this task allows to separately study motor preparation and motor execution, as the sequence is indicated at the start of a trial and only has to be executed after a subsequent Go signal. Sobierajewicz et al. (2016) used this paradigm to study motor imagery by employing sequences in a practice phase that had to be executed, imagined, or withheld after the Go/NoGo signal. In a final test phase, when all sequences had to be executed, learning effects were observed for previously imagined sequences. This paradigm also allows to derive event-related lateralized (ERL) measures from the EEG above motor areas, which seem highly specific for motor-related processes (Galdo-Álvarez & Carrillo-de-la-Peña, 2004; Kranczioch, Mathews, Dean, & Sterr, 2009). Sobierajewicz et al. (2016) observed ERLs with a maximum above motor areas after the Go/NoGo signal in the practice phase that were comparable for imagined and executed sequences, while they differed from sequences that had to be withheld. These results suggest that participants used motor imagery rather than visual imagery in the case of mental simulation. ERLs can also be used to examine the preparation phase before the Go/NoGo signal. For example, De Kleine and Van der Lubbe observed no differences between familiar and unfamiliar sequences for ERLs above hand-motor areas during the preparation phase. At the same time, above visual areas, more lateralized activity-that is, the contralateral delay activity (CDA; Klaver, Talsma, Wijers, Heinze, & Mulder, 1999), was present in the case of preparing new sequences. This effect may be due to the involvement of visual short-term memory (VSTM) and suggests that in this case participants used visual imagery while preparing an unfamiliar sequence. 

 The major issue to be addressed in the current study is to what extent motor imagery can replace physical practice while learning the execution of a fine motor skill. This issue was addressed by employing the aforementioned Go/NoGo DSP paradigm. For the same participant, different sequences were used of which the Proportion of Execution versus imagined trials was varied during a practice phase. By comparing performance on the different sequences in a subsequent test phase, we may establish the extent to which learning by motor imagery can replace physical practice. The employed within-subject design implies increased sensitivity in establishing the required extent of physical training for an optimal learning effect as compared to the studies by Allami et al. (2008, 2014). The second issue to be addressed is whether the explicit instruction to perform motor imagery is required to obtain learning effects. Therefore, a group of participants was included that was instructed to withhold executing the sequence rather than to perform motor imagery during the practice phase. We measured EEG to derive ERLs but limited this to the final test phase. We were interested whether the preparation of a movement sequence depended on the Proportion of Execution/motor imagery during the practice phase. Although motor preparation, reflected in ERLs above motor areas, need not depend on practice (see De Kleine & Van der Lubbe, 2011), the amount of activity as measured with the CDA might be different for sequences that had less or no physical practice as there may be more involvement of VSTM. We predicted effects to be more pronounced for the control group, as learning effects were expected to be smaller in the case of no instruction to perform motor imagery. 

## Method

### Participants

 Twenty-four participants (seven male, 17 female) took part in this experiment who reported to have no history of mental or neurological disorders. They were recruited from the local student population at the Adam Mickiewicz University. All participants were aged between 20 and 30 years (*M*_age_ = 23.1, *SD* = 2.19). Informed consent was obtained from each participant prior to the experiment. Participants were also requested to complete Annett`s Handedness Inventory (Annett, 1970). Twenty-one of them were assessed to be right-handed, and three of them were left-handed. 

### Stimuli and task

**Figure 1.**
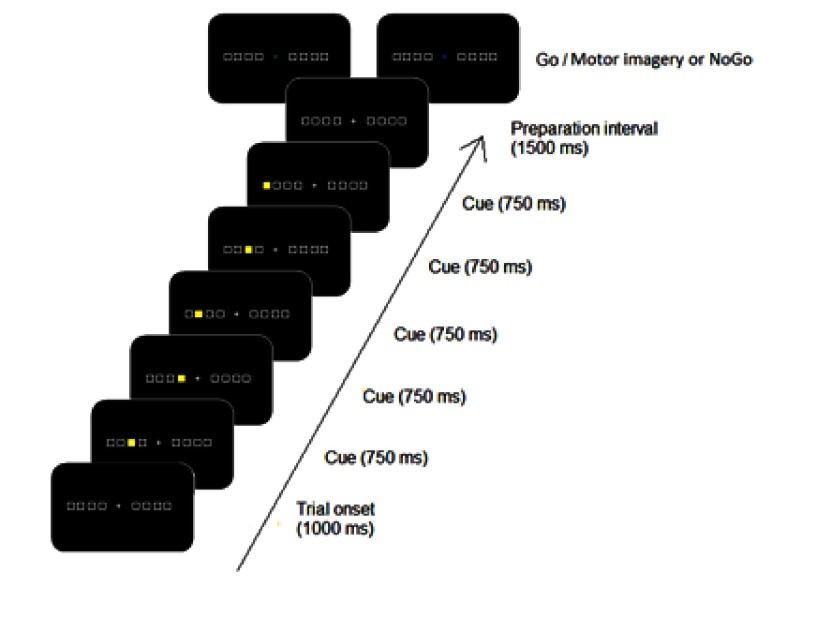
An overview of the presented stimuli in the Go/NoGo DSP task. After presentation of the stimulus sequence either a green cross (here, top left) or a blue cross (top right) was presented. For the motor imagery and the control group, a green cross implied that the sequence had to be executed (Go). For the motor imagery group, the blue cross indicated that execution of the sequence had to be mentally imagined. For the control group, the blue cross implied that no responses should be given (NoGo).

An overview of the sequence of stimuli on a trial is displayed in Figure 1. Stimuli were displayed on a CRT monitor with a refresh rate of 60 Hz. Each trial started with a beep of 300 Hz for 300 ms. Next, a fixation cross (1.3°) was presented in the center of the screen together with eight horizontally aligned squares (2.5°)—four on the left and four on the right side of the fixation cross. The alignment of the eight stimulus squares had a total visual angle of 26.5°. The eight squares and fixation cross were drawn with a grey color line on a black background. After 1,000 ms one of the squares was colored yellow for 750 ms, a second square was colored yellow, and so forth, until a fifth square was colored yellow. The coloring of the squares within a trial always occurred on either the right or the left side. After a time interval of 1,500 ms relative to filling the last square, a response cue was presented at fixation, either in green or in blue. In the case of a green cross (Go), the presented sequence had to be reproduced by pressing the five corresponding buttons, whereas after a blue cross, the sequence had to be mentally executed (i.e., motor imagery) or withheld (i.e., motor inhibition).

### Design and Procedure

At the start of the experiment, participants first received oral instructions. They were told to sit comfortably in a dimly lit room in front of a computer screen. Participants placed their little finger, ring finger, middle finger, and index finger of their left and right hands respectively on the *a*, *s*, *d*, *f* and the *;*, *l*, *k*, *j* keys of a computer QWERTY keyboard. The monitor was placed right in front of the participant at a distance of 70 cm. Participants were instructed to respond as fast and accurately as possible, and they were requested to focus on the fixation cross during the presentation of the sequence and while carrying out the task. Feedback about incorrect responses (“incorrect response” was displayed on the screen) was presented when a participant pressed the button before the Go/NoGo signal or when a false button press was made. Halfway through each block and after each block, a pause was provided in which the participants could relax. After each block, participants were shown their mean response times (RTs) and percentage of correct responses (PC).

Participants were divided into two groups. In the first group, they were instructed to imagine executing the sequence in the case of a NoGo signal (the motor imagery group), while in the second group they were simply instructed to withhold their responses after a NoGo signal (the control group). In the motor imagery group, participants were asked to simulate a movement from a first-person perspective (i.e., to imagine the sensation of executing a sequence). They were instructed to “feel” a movement by explaining the difference between visual versus motor imagery (“imagine yourself walking on the street-you can see yourself walking” vs. “imagine as if you are walking-you imagine your movements during walking”). Moreover, they were asked to imagine only a movement and not a sequence of numbers, symbols, or sounds.

All participants took part in the experiment on three consecutive days. On the first day, they signed an informed consent form and took part in a practice phase of six blocks consisting of 120 sequences. On the second day, they also performed six practice blocks consisting of 120 sequences. On the third day, they took part in the test phase, which consisted of six blocks with 120 sequences. On each day, participants were tested for about 2 hr 30 min.

During the practice phase, each block consisted of five different types of sequences which had to be executed with different probabilities. In the motor imagery group, 24 sequences of type A had to be imagined (0% execution, 100% motor imagery); six sequences of another type B had to be executed (25% execution) meaning 18 sequences of type B had to be imagined; 12 sequences of type C had to be executed meaning 12 imagined (50% execution); 18 sequences of another type D had to be executed (75% execution) meaning six sequences of type D had to be imagined; finally, 24 sequences of type E had to be executed (100% execution). Each block contained 120 sequences. In the control group, participants had the same proportions of execution trials, but were instructed to simply withhold the movement on NoGo trials.

During the test phase the same sequences were used as during the practice phase, but now both groups had to execute all sequences. Furthermore, in the test phase, EEG was measured to investigate differences in motor preparation and the possible involvement of VSTM for the different types of sequences.

In the experiment, six different structures of movement sequences (always consisting of five finger movements) were created with four (1 to 4) possible response options: 12432, 13423, 14213, 13241, 14312 and 21431. For each structure four different versions of sequences were used by assigning each of four fingers (little finger, ring finger, middle finger, index finger) to the four response options. The different employed sequences are shown in Appendix A. Use of this procedure should eliminate finger-specific effects, and also should have the consequence that all sequences had the same level of complexity for each participant. The sequences for the types A to E (see above) were counterbalanced across participants and fingers.

### Recording and Data Processing

 EEG was recorded during the test phase from 64 active channels with a sampling rate of 1,000 Hz. Electrodes were placed on an ActiCap (Brain Products, GmbH) at appropriate locations according to the extended International 10-20 system (Oostenveld & Praamstra, 2001). An average reference was used, which was built into the amplifier (QuickAmp, Brain Products, GmbH). A ground electrode was located at the Fpz location. The resistance between electrode and skin was less than 5 kΩ. Electrooculographic (EOG) activity was recorded bipolarly, both vertically (vEOG) from above and below the right eye and horizontally (hEOG) from the outer canthi of both eyes. Electromyographic (EMG) activity was measured bipolarly by attaching EMG electrodes on the musculus flexor digitorum superficalis and on the processus styloideus ulnae of the right and left hands. 

EEG, EOG, and EMG data plus markers signaling stimulus events, responses, and block-relevant information were registered with Vision Recorder (Brain Products-version 2.0.3). Offline, analyses were performed with Brain Vision Analyzer (version 2.0.4) software. First, data were low-pass filtered (30 Hz), segments were selected from -2,500 ms to 4,000 ms relative to the Go/NoGo signal while a baseline was set from -1,600 ms to -1,500 ms. We were especially interested in the 1,500 ms time interval between the offset of the last presented stimulus and the Go/NoGo signal as motor preparation and/or the involvement of VSTM was thought to take place during this interval. We excluded trials with major artifacts from further analyses (i.e., the maximum allowed voltage step was 100 µV/ms, minimum/maximum allowed amplitude was -/+ 150 µV, and lowest allowed activity difference within 50 ms intervals was 0.5 µV). Ocular correction was carried out with the semiautomatic independent component analysis (ICA) algorithm. We computed the averages for each type of task per hand, which enabled us to determine event-related lateralized (ERL) measures (detailed later).

### Behavioral Parameters

 RT was defined as the time between the onset of the Go signal and depression of the first key, and as the time between two consecutive key presses within a sequence ( De Kleine & Van der Lubbe, 2011; Ruitenberg, De Kleine, Van der Lubbe, Verwey, & Abrahamse, 2011). As the total number of levels in our experiment was relatively high, we decided to reduce the number of levels of the variable key from five to two, where level 1 corresponds to key 1, while level 2 corresponds to the average of keys 2 to 5. With the reduction from five to two levels, we were still able to distinguish between initiation time and execution time of the remaining sequence. For the practice phase, mean RTs were evaluated statistically by repeated-measures analysis of variance (ANOVA), with Block (12), Proportion of Execution (4), and Key (2) as within-subject factors, and Group (2) as between-subjects factor. For the test phase, mean RTs were evaluated with the same factors, but Block and Proportion of Execution had now six and five levels, respectively, as responses were required to all sequences in the six test blocks. 

 Analyses on PCs were carried out after performing an arcsine transformation to stabilize variances (Abrahamse & Verwey, 2008). A repeated-measures ANOVA was run for the practice phase, with Group (2), Block (12) and Proportion of Execution (4) as independent variables; and for the test phase, with Group (2), Block (6) and Proportion of Execution (5) as independent variables. 

### EEG Measures

 The ERLs were computed for the test phase as we wanted to investigate motor preparation and/or the involvement of VSTM after motor skill learning with different proportions of executed/imagined/withheld sequences. ERLs were calculated for frontal, frontocentral, central, centroparietal, parietal, and occipital electrodes. The ERL method can be considered as an extension of the procedure used to compute the lateralized readiness potential (LRP), which is only calculated for central electrodes (De Jong, Wierda, & Mulder, 1988; Gratton, Coles, Sirevaag, Eriksen, & Donchin, 1988). ERLs are based on a double subtraction procedure performed on ERPs for left hand (LH) and right hand (RH) trials, which extracts the activity that is specific to the relevant side. They are computed in the following way: 

(1) $$ERL=\frac{\left(LH\left(contra-ipsi\right)+RH\left(contra-ipsi\right)\right)}{2}$$

Lateralized activity was determined in 250 ms intervals from stimulus offset (-1,500 ms) until the Go/NoGo signal. For each of three electrode pairs (C1/2, P3/4, and PO7/O8), ERLs were analyzed by a repeated-measures ANOVA performed with the variables Group (2), Time window, (6) and Proportion of Execution (5). The electrode pairs were chosen on the basis of inspection of the current results and the results in the study of De Kleine and Van der Lubbe (2011). 

### EMG

 EMG activity was measured bipolarly to assess muscular activity during the practice phase in order to control whether participants did not flex their muscles during motor imagery and motor inhibition. The extent of motor activation was determined by performing a wavelet analysis on the raw signal. First, the EMG signal was low-pass filtered at 50 Hz (24 dB/oct) and high-pass filtered at 20 Hz (24 dB/oct). A complex Morlet wavelet was chosen (*c* = 5), with the lower and upper boundaries for the extracted layer set at 20 and 50 Hz, respectively (Carillo-de-la-Peña, Galdo-Álvarez, & Lastra-Barreira, 2008). 

The obtained power of the EMG was analyzed with Group (2), Block (12), EMG-channel (2), Hand (2), and Task (Go or NoGo; 2) as variables. We examined the time window from 1,000 ms until 5,000 ms after the Go/NoGo signal, as this was the time interval during which the sequence had to be executed, imagined, or withheld.

All statistical analyses of our behavioral, EEG, and EMG measures were performed with SPSS (IBM Statistics SPSS 22). Greenhouse-Geisser ε correction was applied to the analyses whenever appropriate. To increase sensitivity for detecting gradual differences as a function of Block and Proportion of Execution we examined linear, quadratic, and cubic contrasts.

## Results

### Practice Phase

**Figure 2.**
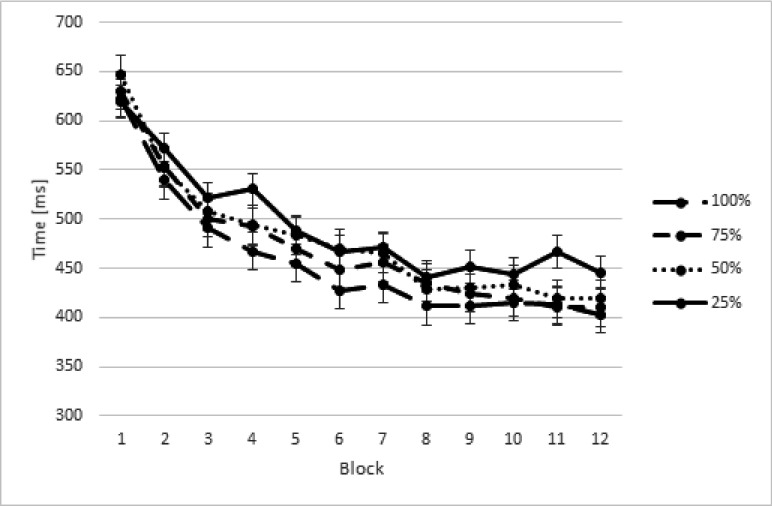
Response times (RTs) in milliseconds (ms) in the training phase for both groups as a function of different proportion of executed sequences. Error bars represent standard errors.

An ANOVA with repeated-measures on RTs was performed with the following within-subject variables, Block (12), Key (2), Proportion of Execution (4), and the between-subjects variable Group (2). An overview of the RTs averaged across keys is displayed in Figure 2.

**Figure 3.**
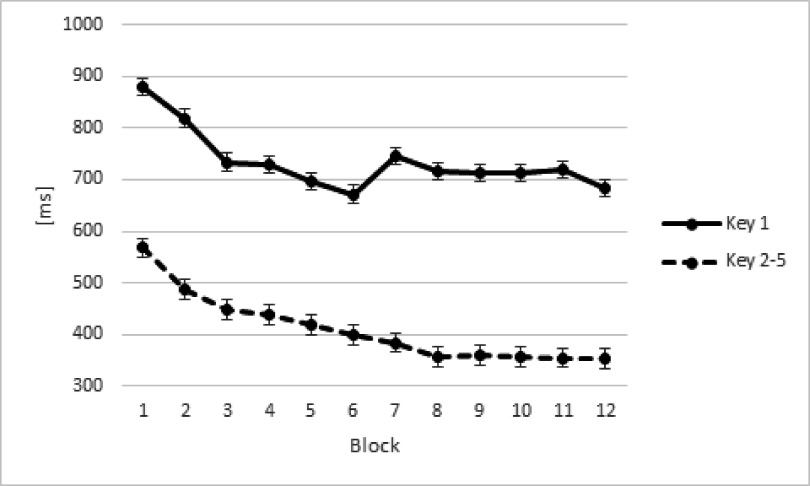
Response times (RTs) in milliseconds (ms) in the training phase for both groups according to Key. Error bars represent standard errors.

A main effect of Block was observed, *F*(11, 24) = 21.77, ε = .27, *p* < .001, η_p_^2^ = .50. Contrast analyses revealed a linear trend, *F*(1, 22) = 33.28, *p* < .001, a quadratic trend, *F*(1, 22) = 42.72, *p* < .001, and a cubic trend, *F*(1, 22) = 8.54, *p* < .008. Figure 3 suggests that these effects reflected a general decrease in RT during practice, although this decrease seems to be stronger in the initial learning phase. A main effect of Key was observed, *F*(1, 22) = 103.15, *p* < .001, η_p_^2^ = .82, revealing that the average RT for keys 2 to 5 was smaller than for key 1 (mean RTs for keys 1 to 5 for the motor imagery group were 747, 410, 428, 426, and 327 ms, respectively; mean RTs for keys 1 to 5 for the control group were 724, 422, 438, 456, and 377 ms, respectively). An effect of Proportion of Execution was observed, *F*(3, 66) = 5.76, ε = .64, *p* < .007, η_p_^2^ = .21. Contrast analyses showed a linear trend, *F*(1, 22) = 14.07, *p* < .001. These results indicated that the more participants physically executed the sequences, the faster were their RTs (see Figure 2). The Block × Proportion of Execution interaction was not significant, *p* = .23. No significant Group × Block interaction, *p* = .77, and no significant Group × Proportion of Execution interaction was observed, *p* = .70. These results indicate that in both groups, participants executed the sequences equally fast, despite of the different instructions (imagery vs. no execution) with regard to the NoGo trials during the practice phase. Results also revealed no significant Group × Key interaction, *p* = .46. RT results also revealed a Block × Key interaction, *F*(11, 24) = 3.24, ε = .34, *p* < .001, η_p_^2^ = .13, cubic trend, *F*(1, 22) = 8.23, *p* = .009. For key 1, we observed an increase in initiation time of a motor response after the sixth block, which reflects the beginning of the second practice day (see Figure 3). To assess whether this interaction reflects a stronger Block effect for the average of keys 2 to 5 than for key 1, we performed an additional analysis excluding the results from the 7th block. An effect of Key was observed, *F*(1, 22) = 99.51, *p* < .001, η_p_^2^ = .82, linear trend, *F*(1, 22) = 99.51, *p* < .001. These results also revealed a significant Block × Key interaction, *F*(10, 22) = 3.01, ε = .35, *p* = .001, η_p_^2^ = .12, cubic trend, *F*(1, 22) = 6.23, *p* = .02, showing that the time needed to execute the sequences (the average of keys 2 to 5) becomes faster with practice relative to the decrease in response initiation time.

**Figure 4.**
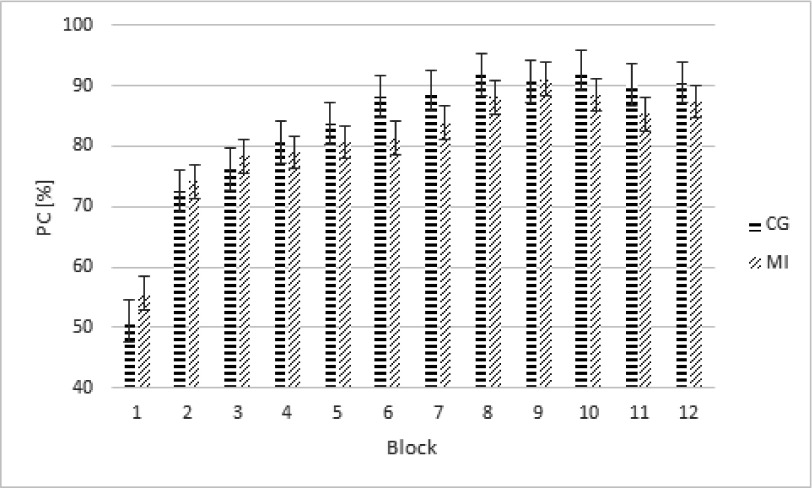
Percentages of correct responses (PCs) of the averages of to be executed sequences in the training phase for the motor imagery group (MI) and the control group (CG). Error bars represent standard errors.

A repeated-measures ANOVA was performed on arcsine transformed error percentages as a function of Group (2), Block (12), and Proportion of Execution (4). The analysis did not reveal significant differences between groups as a function of Proportion of Execution, *p* = .44. These results suggest that there are no differences in accuracy between the groups and between the different proportions of execution. A significant difference was observed as a function of Block, *F*(11, 24) = 24.56, ε = .33, *p* < .001, η_p_^2^ = .53, linear trend, *F*(1, 22) = 37.74, *p* < .001; quadratic trend, *F*(1, 22) = 55.93, p < .001; (see Figure 4). These results indicate that the number of correct responses increased with practice, while this effect was most prominent in the earlier blocks.

### Test Phase

For the test phase an ANOVA with repeated measures was performed, with variables Group (2), Block (6), Key (2), and Proportion of Execution (5). Results revealed no significant group differences, *p* = .64.

**Figure 5.**
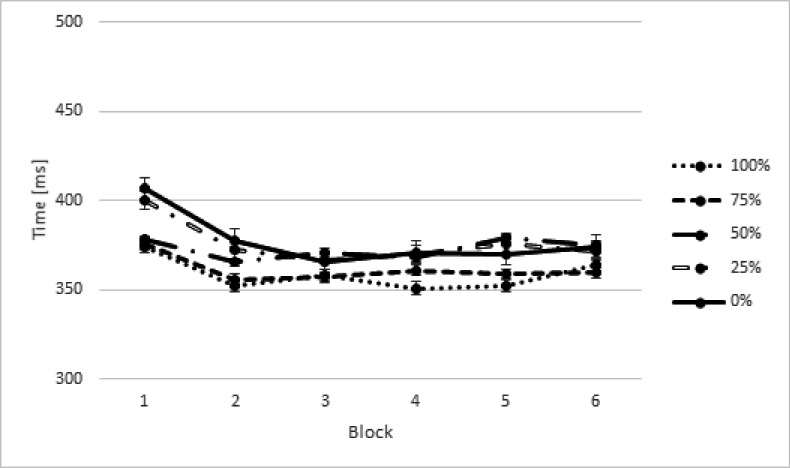
Response times (RTs) in milliseconds (ms) in the test phase for the motor imagery group and the control group according to different percentage of executed proportion. Error bars represent standard errors.

A significant difference of RTs was observed as a function of Key, *F*(1, 22) = 75.97, *p* < .001, η_p_^2^ = .78, which again revealed that RTs for key 1 were slower than for the average of keys 2 to 5. A significant difference was also observed as a function of Proportion of Execution, *F*(4, 88) = 3.26, ε = .81, *p* < .02, η_p_^2^ = .13, linear trend, *F*(1, 22) = 8.48, *p* = .008, showing that the more participants physically practiced, the less time they needed to produce the correct movement sequence (see Figure 5).

Separate *t*-tests revealed that sequences which were practiced with a proportion of 0% execution, 25%, and 50% in the practice phase were executed slower than sequences that were executed on 100% of the trials in the practice phase, *t*(23) > 2.73, *p* < .01. These results indicate that participants had to execute sequences at least with a proportion of 75% to obtain similar learning effects as compared to 100% execution.

**Figure 6.**
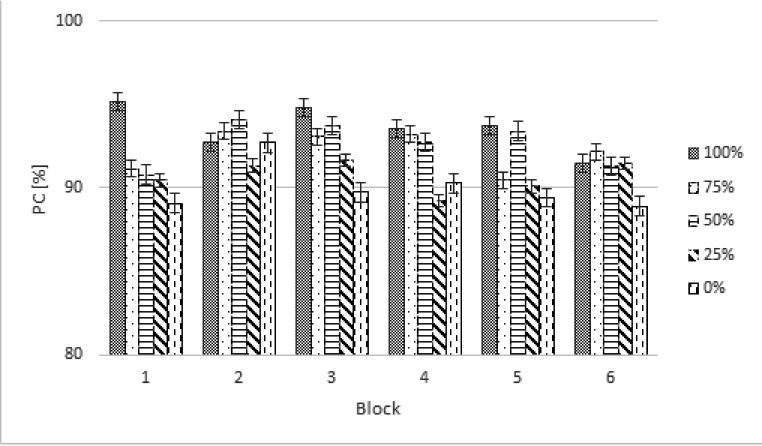
Percentages of correct response (PCs) in percentages (%) in the test phase for motor imagery (MI) group and control group (CG). Error bars represent standard errors.

In the test phase, a similar repeated-measures ANOVA was performed on arcsine transformed error percentages, with variables Group (2), Proportion of Execution (5), and Block (6). Results showed no significant group differences, *p* = .65 (see Figure 6). The analysis also revealed no differences between groups as a function of Proportion of Execution, *p* = .18, linear trend, *F*(1, 22) = 3.18, *p* = .09. Separate *t*-tests revealed no significant differences as a function of Proportion of Execution, *t*(23) > -0.54, *p* < .17.

### EEG Results for the Test Phase

**Figure 7.**
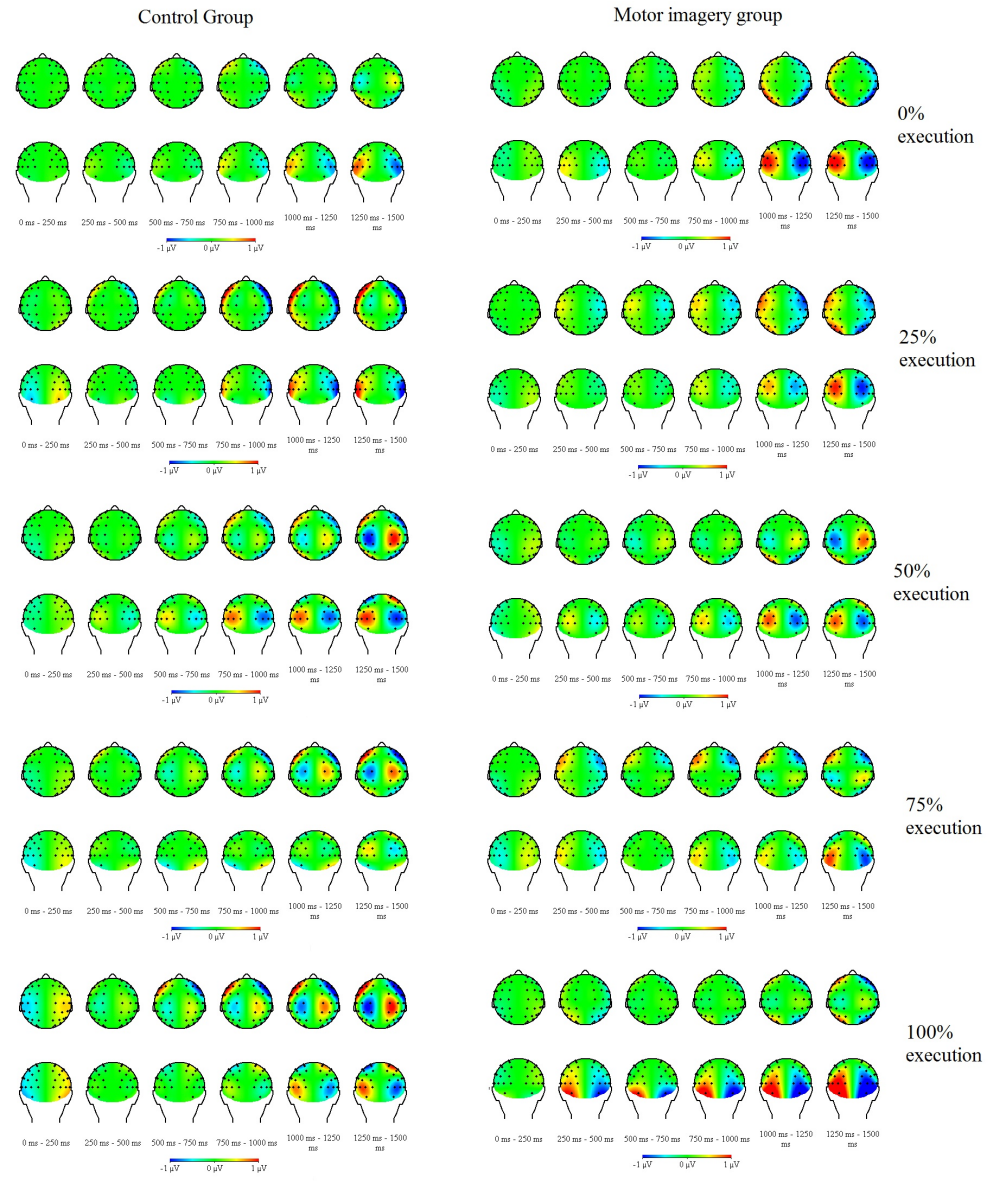
Topography of event related lateralizations (ERL s) for the different proportions of motor execution and motor imagery in the motor imagery group, and of motor execution and motor inhibition in the control group in the test phase, from -1,500 ms to Go/NoGo signal (0 ms). The left side of the brain displays the contra-ipsilateral difference. Negativity on the left hemisphere implies that activity was more negative on contralateral than on ipsilateral electrodes, which is visible for both groups: the control group and the motor imagery group.

Topographical maps for activity from the offset of the last stimulus (0 ms) to the Go/NoGo signal (1,500 ms) are displayed in Figure 7, averaged across each level of Proportion of Execution and Group, from all blocks from the test phase. For each of three selected electrode pairs (C2/1, P4/3, and PO8/7), ANOVAs were performed with the variables Group (2), Time window (6), and Proportion of Execution (5).

#### C2/1 electrode pair

The lateralized activity on the C2/1 electrode pair did not differ between groups, *F*(1, 22) = 2.34, *p* = .14, η_p_^2^ = .1. A significant difference was observed as a function of Time Window, *F*(5, 110) = 11.73, ε = .4, *p* < .001, η_p_^2^ = .35, linear trend, *F*(1, 22) = 13.82, *p* = .001, and quadratic trend, *F*(1, 22) = 10.38, *p* = .004. These results indicated that the lateralized activity changed depending on time, showing an increase of contralateral negativity from -750 ms relative to the Go/NoGo signal. No significant Time Window × Proportion of Execution interaction was observed, *F*(20, 440) = 1.67, ε = .23, *p* = .16, η_p_^2^ = .7. However, contrast analyses revealed a linear × linear trend for the Time Window × Proportion of Execution interaction, *F*(1, 22) = 6.52, *p* < .02, suggesting an increase of negativity on the C2/1 electrode pair over time as a function of Proportion of Execution. A Time Window × Proportion of Execution × Group interaction was not significant, *F*(20, 440) = 0.36, *p* = .86, η_p_^2^ = .2. However, the contrast analysis showed a quadratic × linear trend for Time window, Proportion of Execution, and Group, *F*(1, 22) = 5.27, *p* = .03. Separate analyses were performed for the last time window (from -250 ms to the Go/NoGo signal) as a function of Proportion of Execution and Group. No significant difference was observed as a function of Proportion of Execution, *F*(4, 88) = 2.1, ε = .85, *p* = 0.1, η_p_^2^ = .09, linear trend, *F*(1, 22) = 4.93, *p* < .04. No significant Proportion of Execution × Group interaction was observed, *F*(4, 88) = 0.2, *p* = .97, η_p_^2^ = .004. No other significant effects were observed, p >.14.

#### P4/3 electrode pair

No differences were observed on lateralized parietal activity between the two groups, *p* = .73. A main effect of Time Window was observed, *F*(5, 110) = 9.25, ε = .37, *p* = .001, η_p_^2^ = .3. Trend analyses revealed a significant linear trend, *F*(1, 22) = 10.15, *p* = .004. From -750 ms to Go/NoGo signal, we observed stronger positivity at the P4/3 electrode pair. No other significant effects were observed, p > .86.

#### PO8/7 electrode pair

No significant difference was observed for the lateralized activity at the PO8/7 electrode pair between groups, *F*(1, 22) = 1.53, *p* = 0.23, η_p_^2^ = .07. A main effect of Time Window was observed, *F*(5, 110) = 33.54, ε = .41, *p* < .001, η_p_^2^ = 0.6, linear trend, *F*(1, 22) = 48.14, *p* < .001; cubic trend, *F*(1, 22) = 7.37, *p* = .01. The latter results show that the negativity on this electrode pair decreased with time, and from -750 ms it changed being more and more positive at a different rate. No other significant effects were observed, p > .37.

### EMG

**Figure 8.**
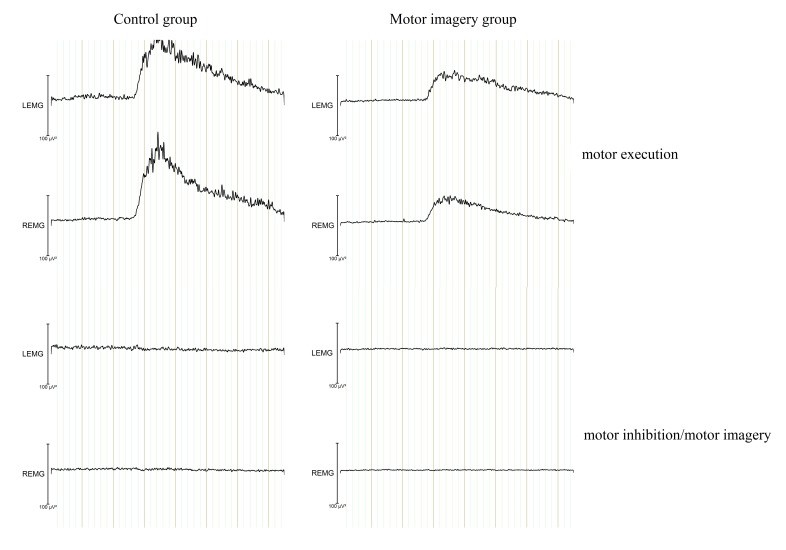
Outcome of the wavelet analysis performed on the raw electromyographic (EMG) signal measured from the electrodes attached to the left and right forearms. The grand averages are presented for the motor imagery/motor inhibition and motor execution from the Go/NoGo signal (0 ms) to 6,000 ms.

Figure 8 shows the EMG signal related to both hands while executing the task. First, we compared EMG during motor execution with EMG during motor imagery and motor inhibition. Figure 8 shows that in both groups the EMG signal was larger for executed sequences as compared to imagined and inhibited sequences. A significant difference was observed as a function of Block, *F*(11, 242) = 3.83, ε = .2, *p* < .03, η_p_^2^ = .15, linear trend, *F*(1, 22) = 5.1, *p* = .03. An EMG-Channel × Hand interaction was observed, *F*(1, 22) = 91.59, *p* < .001, η_p_^2^ = .81. This interaction indicates that muscular activity was larger for the executing hand than for the passive hand during task execution. A significant Block × EMG-Channel × Hand × Condition × Group interaction was observed, *F*(11, 242) = 2.52, ε = .35, *p* = .05, η_p_^2^ = .1, showing a decrease in muscular activity on the EMG-channel with practice in both groups.

We also compared EMG between motor imagery and motor inhibition. Results revealed no difference, *F*(1, 22) = 1.74, *p* = .2, η_p_^2^ = .07. A main effect of Block was observed, *F*(11, 242) = 6.09, ε = .26, *p* = .001, η_p_^2^ = .22, linear trend, *F*(1, 22) = 11.89, *p* = .002. An EMG-Channel × Hand interaction was observed, *F*(1, 22) = 4.29, *p* < .001, η_p_^2^ = .49. This effect seems to indicate that participants activated their muscles at least to some extent during imagining and inhibiting.

In conclusion, the results of our EMG analyses revealed that participants moved their fingers mainly in the case of motor execution although there was some remaining motor activity during motor imagery and motor inhibition.

## Discussion

In the present study, we asked to what extent learning a fine motor skill by motor imagery may substitute physical practice. Furthermore, we were interested to see whether the explicit instruction to perform motor imagery is needed to show sequence-specific learning effects. A Go/NoGo DSP task was employed with different required sequences of finger movements to investigate learning effects through behavioral and electrophsyiological measures. This was done both for a motor imagery and a control group. The experiment was divided into two phases: a practice phase in which different sequences were employed of which the Proportion of Execution and motor imagery/no execution was varied, and a test phase in which all sequences had to be executed in order to assess differential learning effects. We used a control group that simply was instructed to withhold executing the sequence on different proportions of trials in the practice phase to establish whether observed learning effects are really due to an explicit motor imagery instruction. First, we will address the question to what extent learning a fine motor skill by motor imagery may replace learning by physical practice. Second, we will focus on the specificity of the effect of motor imagery by comparing performance between both groups.

 Behavioral results in the practice phase showed that the more participants physically executed the sequences, the faster were their RTs. A main effect of Key indicates that the time required to initiate the response was longer than the average time needed to execute the remaining response sequence (e.g., see Abrahamse, Ruitenberg, De Kleine, & Verwey, 2013). A significant Block × Key interaction indicates that execution time changes more due to practice than the time needed to initiate executing a sequence. We observed no differences in accuracy between the different Proportions of Execution. In the crucial test phase, analyses of RTs revealed that participants had to execute sequences at least with a proportion of 75% in the practice phase to achieve similar learning effects as by full physical practice. Sequences with lower Proportions of Execution were performed slower, which implies that they induced reduced learning effects. Importantly, no effect of Proportion of Execution was observed on accuracy in the test phase. Thus, accuracy of executing a sequence in the test phase that was not executed before was equally good as compared to a sequence that was fully practiced before. This observation suggests that motor imagery induces motor learning, although reaching a maximal speed of execution seems to require at least 75% of physical practice. Hence, learning a fine hand motor skill by motor execution cannot be fully replaced by learning with motor imagery. A possible reason why learning with motor imagery cannot fully substitute learning with motor execution is the lack of proprioceptive feedback in the case of motor imagery. This idea corresponds with the view advocated by Gentili et al. (2010, 2006). In this respect, the concept of internal models, which constitute a system that represents the behavior of a natural process and is associated with motor control, seems relevant (Gentili et al., 2010; Wolpert & Miall, 1996). It is thought that during mental training the state estimation (i.e., sensorimotor state, which is related to position, proprioception, velocity, etc.) is based only on forward internal model output, while during physical practice this output is combined with sensory feedback (Gentili & Papaxanthis, 2015; Wolpert, Ghahramani, & Jordan, 1995). As a consequence, state estimation during physical practice is more accurate and precise. Nevertheless, according to hierarchical models, motor imagery leads to a reinforcement of the structure of motor representation at the cognitive level, and thereby to increased expertise (di Rienzo et al., 2015; Frank, Land, Popp, & Schack, 2014; Jeannerod, 1995; Schack, 2004). This may be the reason why participants in the test phase became as accurate but not as fast in their performance. Interesingly, in our previous study, we revealed that sequences that were imagined in a practice phase were executed slower and less accurate in the test phase than sequences that were fully executed in the practice phase (Sobierajewicz et al., 2016). In the present study, participants had much more practice (i.e., two days) as compared to our previous study, in which participants practiced only on a single day (i.e., about 2 hr). Thus, learning effects (especially with regard to accuracy), seem to be affected by the duration of practicing motor imagery. However, Sobierajewicz et al. (2016) also revealed that imagined sequences were nevertheless executed faster and more accurate than novel sequences (not included here), which demonstrates that motor imagery induced the learning of a fine hand motor skill. 

Our second question was whether the explicit instruction to perform motor imagery is really needed to show sequence-specific learning effects. To do so, we employed two groups of participants with different instructions—the motor imagery group had to imagine carrying out a movement after a NoGo signal from a first person perspective, while the control group was instructed to withhold the movement after a NoGo signal. To our surprise, we observed indistinguishable learning effects in both groups. Results reflected a general decrease in RT and an increase in PC with practice in both groups. Moreover, the test phase also revealed no group differences in RT and PC. Hence, the sequences were carried out as accurately as possible in both groups, despite of the instructed mode in the practice phase. Similar learning effects can be related to the presence of motor preparation in both groups, as all participants had to prepare a movement until the Go/NoGo signal. This finding raises the question whether motor imagery might be equivalent to motor preparation. Participants in the control group could also imagine a movement during preparation intervals, and in that time they could also mentally practice a sequence. Even though they did not receive an explicit instruction to imagine a movement, they could do this while waiting for a Go/NoGo signal.

 We also examined ERLs to assess the effect of Proportion of Execution and motor imagery/no execution on motor preparation during the final test phase. No main effect of Proportion of Execution was observed on the activity on the central electrode pair (C2/C1) and on the activity on the parietal and the occipital electrode pairs (P4/P3, PO8/PO7), showing similar activity during motor preparation. Results revealed more negativity just before the Go/NoGo signal on the central electrode pair for the control group. Furthermore, stronger activity on the occipital electrode pair might reflect the additional involvement of VSTM during motor preparation in both groups. These results seem consistent with a previous study of De Kleine and Van der Lubbe (2011). In their study, it was observed that unfamiliar sequences also seem to require visual memory, thus, imagined sequences seem in some way comparable to unfamiliar sequences. Furthermore, on the basis of both behavioral and EEG results we suggest that visual presentation of the stimuli and memorizing the sequence already induced motor skill learning. This notion can also explain why a similar lateralized activity was observed during motor preparation in both groups despite of the way of learning in the practice phase. 

 Our study did not demonstrate results identical to the study of Allami et al. (2008), as we still observed that the speed of performance was higher after full motor execution relative to 75% execution. This result was observed for both groups, while in the study of Allami et al. (2008) groups with at least 50% trials of imagery displayed similar performance as full motor execution. Several reasons may be given for this discrepancy. Effects are likely related to the type of motor task. Learning a fine motor skill may require other and more subtle spatial skills than performing a grasping task. Furthermore, in the study of Allami et al. (2008), there was no explicit preparation phase, in which participants could imagine or prepare the movement. In addition, we used a within-subject design, which seems more sensitive in detecting differences than a between-subjects design. Finally, the task chosen in our study allows to separate sequence-specific from unspecific learning. Hence, effects observed in other studies might possibly reflect unspecific learning due to an increased familiarity with the task, instead of sequence-specific learning. 

 In conclusion, our results revealed that a combination of mental imagery with a high rate of motor execution may be beneficial for the learning of a fine motor skill (especially with regard to its accuracy). However, the role of mere motor preparation for learning a fine motor skill should be specified in future research. These results have relevant consequences for therapies using mental practice, for example, for children with cerebral palsy or patients after stroke (Jongsma et al., 2015; Kim & Lee, 2015). Our results showed that motor imagery may especially help to improve the accuracy of the motor skill, whereas physical practice will be needed to improve response speed. 

## Author Note

This work was supported by grant UMO-2012/05/B/HS6/03806 from the National Science Centre in Poland. Rob van der Lubbe was additionally supported by the Institute for Biomedical Technology and Technical Medicine (MIRA). We thank Joanna Minge for her help with data collection. The study was approved by the local ethics committee of the Adam Mickiewicz University and was performed in accordance with the Declaration of Helsinki. The authors declare no conflicts of interest concerning the content of this article.

## Appendix A

Sequences of Five Key Presses used in the Experiment.

6 structures of the sequence, 4 versions each

1-a, 2-s, 3-d, 4-f

1-;, 2-l, 3-k, 4-j
